# Interdisciplinary Stroke Recovery Research: The Perspective of Occupational Therapists in Acute Care

**DOI:** 10.3389/fneur.2019.01327

**Published:** 2019-12-17

**Authors:** Jessica Ranford, Jessica Asiello, Alison Cloutier, Kimberly Cortina, Helena Thorne, Kimberly S. Erler, Natasha Frazier, Caitlin Sadlak, Abigail Rude, David J. Lin

**Affiliations:** ^1^Department of Occupational Therapy, Massachusetts General Hospital, Boston, MA, United States; ^2^Center for Neurotechnology and Neurorecovery, Department of Neurology, Massachusetts General Hospital, Harvard Medical School, Boston, MA, United States; ^3^Department of Occupational Therapy, MGH Institute of Health Professions, Boston, MA, United States

**Keywords:** occupational therapy, stroke recovery, acute care, neurorehabilitation, participation

## Abstract

As acute stroke treatments advance, more people survive the initial stroke event and live with long-term neurological impairments that impact functional outcomes and quality of life. In accordance with International Classification of Functioning (ICF), living with long-term neurological impairments can limit survivors' activity performance and restrict participation in valued life roles and routines. Research focused on longitudinal analysis of functional measures and outcomes after stroke are critical for determining early indicators of long-term participation and quality of life and guiding rehabilitation resource allocation. As core members of the interdisciplinary stroke recovery treatment team throughout the post-acute care continuum, occupational therapists (OTs) directly address stroke survivors' ability to participate in meaningful daily activities to promote function and quality of life. Just as in clinical care in which multidisciplinary, team-based perspectives are vital, OTs provide invaluable perspectives for stroke recovery research. Here we describe OTs' role in a collaborative, interdisciplinary research study aimed at comprehensively understanding upper extremity motor recovery after stroke and its impact on individuals across the post-acute care continuum. This article discusses the importance of the OTs' perspectives in conducting interdisciplinary, longitudinal stroke recovery research. The challenges, strategies and recommendations for future directions of advancing the role of OTs in multidisciplinary stroke recovery research are highlighted. We use this perspective as a call to action to the stroke recovery field to incorporate OTs as members of the research team and for OTs to provide their perspectives on ongoing stroke recovery research.

## Introduction

Despite continuous advances in acute interventions, stroke remains the leading cause of disability worldwide (1). Given the aging population and increased rates of survival, the global burden of stroke is expected to continue to increase in the coming years. Although the World Health Organization International Classification of Functioning (ICF) identifies impairments, activity limitations and participation restrictions as components of functioning and disability in the setting of a health condition, much of stroke recovery research occurs in silos (2, 3). All too often research studies are focused only on one axis of the ICF or are restricted to one professional perspective— the neurologist, the rehabilitation scientist, the therapist, etc. There is limited attention toward the interconnectedness of impairment, activity and participation in stroke recovery research. A multidisciplinary approach to stroke research broadens the lens with which we view recovery and provides a multidisciplinary context from which interdisciplinary communication can occur.

The clinical care of patients with stroke employs an interdisciplinary approach. This approach should also be emphasized in stroke research, starting in the acute care setting and across the post-acute care continuum. Occupational therapists (OTs) are essential members of the interdisciplinary team who address all domains of the ICF after stroke across the continuum of care. The overarching goal of occupational therapy is to improve a stroke survivor's ability to engage in meaningful activities, promoting function and quality of life (4). Here we discuss the role of OTs in interprofessional stroke recovery research with a focus on the acute care setting.

This perspective discusses the feasibility of conducting stroke recovery research through the lens of an inpatient occupational therapy department. We highlight the example of clinical OTs (full-time clinicians) in a busy academic, acute care, clinical department participating in stroke-recovery research and becoming integral members of a clinical-research study team. The context of this research, challenges, strategies, and recommendations for future directions are highlighted.

## Context

### Stroke Motor Rehabilitation and Recovery Study

The Stroke Motor Rehabilitation and Recovery Study (SMaHRT) is a neurology, physician-scientist led longitudinal, single site study aimed at understanding the natural history of upper extremity motor recovery after ischemic stroke. Participants are enrolled during their acute stroke hospitalization at Massachusetts General Hospital (MGH) in Boston, MA with the aim of rigorously examining the behavioral, neuroanatomic, and neurophysiologic underpinnings of recovery. The ultimate goal is to develop personalized strategies and neuro-technologies to facilitate neurorehabilitation and enable better recovery for our patients. Launched in June 2017, OTs on the inpatient service at MGH participated in study design and became an integral part of this interdisciplinary research team. The team of OTs perform standardized clinical assessments in the acute care setting as baseline measures. These assessments were paired with longitudinal follow-up in a neurologist-led integrated clinical research outpatient clinic at 6 weeks, 3 months, 6 months and 1-year post stroke. All participants in the study provided written informed consent. The Institutional Review Board at Partners Healthcare approved the study.

### Comprehensive Stroke Center

MGH is recognized as a Comprehensive Stroke Center by the Joint Commission on Accreditation of Healthcare Organizations (JCAHO) for its specialized ability to treat the most complex stroke patients. The MGH Inpatient Occupational Therapy Department leadership team includes a clinical director and four clinical specialists with advanced knowledge and experience in specialized areas of clinical care. The clinical specialists oversee clinical practice, support professional development amongst staff and perform quality process improvement initiatives. There are 28 licensed OTs with 6 assigned to the neurology units (i.e., the neuroscience intensive care unit and two neuroscience hospital floors). OTs rotate through neurology units every 12–15 months and work closely with the occupational therapy clinical specialist in neurology to develop expertise related to this population. In addition to the licensed clinicians, the department also includes one rehabilitation aide who assists with hands-on support and environmental set up for occupational therapy interventions.

### Interdisciplinary Approach to Research

The physician-scientist investigators included occupational and physical therapy leadership in the design phase of this study to select appropriate assessments. The study was initially focused on arm motor impairment but, with perspectives of the occupational therapy team, the aims were refocused to understand how arm impairment influences activity and participation restrictions. Qualifications for OTs to act as study staff included completion of a rotation on the neurology service, commitment to a 2-year time period of data collection, maintenance of typical clinical caseload and productivity expectations, and participation in study trainings. Six OTs were selected to perform inpatient testing. OTs who administered assessments to study participants were sometimes involved in the clinical care of the study participants but did not bill for the study visit since it was not considered standard clinical care.

### Research Test Selection

A series of meetings were held between physicians and occupational and physical therapists to decide on the best research tests to capture not only arm impairment but also activity limitations and participation restrictions. Decisions regarding test selection were based on goals of the study, commonly accepted research measures, clinical relevance, psychometric properties, practicality, and time to complete. Each assessment needed to be administered in an efficient timeframe on an inpatient acute hospital unit with minimal equipment. The interdisciplinary research group selected the final battery of assessments (Table 1). The OTs in the study played a critical role in determining final test battery selection. The MGH Occupational Therapy department advocated to serve as the primary data collectors during the acute stroke inpatient stay. Their clinical expertise in upper extremity motor function, familiarity with several of the assessments and background training in supporting patient performance via environmental set up were key in this regard. The entire test battery was anticipated to take a maximum of 1 h to complete. All research OTs participated in standardized training to ensure inter-rater reliability. Standard operating procedures with written scripts and visual illustrations were developed and used for the assessments in order to ensure assessors' fidelity to standardized testing procedures.

**Table 1:** Outcome measures administered in MGH Stroke Motor Rehabilitation and Recovery Study (SMaHRT) spanning International Classification of Functioning Domains.

**WHO ICF domain**	**Outcome measure**	**Assessment type**
Structure-function	Fugl-meyer motor assessment	Upper extremity impairment
	Dynamometer	Grip strength
	NIH stroke scale	Overall stroke severity
Activity	Box and blocks	Gross motor function
	9-Hole peg	Fine motor function
	Barthel index	Performance of ADLs/iADLs
	Modified rankin scale	Functional independence
	Timed up and go	Mobility and fall risk
	Gait velocity	Mobility and fall risk
Participation	Stroke impact scale 16 (ADLs/iADLs)	Stroke impact on function
	Patient reported outcome measure (PROMIS-10)	Self-assessment of overall health

### Outcome

One hundred patients with stroke consented and enrolled in SMaHRT between June 2017 and April 2019 and continue to be followed as outpatients for integrated clinical research visits. Of the 100 participants, 55 were assessed by OTs. The first 30 participants were enrolled and tested prior to the OTs receiving formal training on the battery of assessments. Due to time constraints of the inpatient occupational therapy group, another 15 participants' assessments were completed by non-OT members of the research team. The reasons for this included therapist availability for participants enrolled on the weekend or after work hours and time sensitivity related to impending discharge.

### Contributions of Occupational Therapists to Stroke Recovery Research

Ongoing discussions with leaders of the SMaHRT research team have highlighted the critical role of OTs in this ongoing stroke recovery research study. First, with regard to study design, stroke recovery research studies do not often account for how stroke affects the individual comprehensively. In this study, given the involvement of OTs in even the beginning stages of study design, all aspects of the ICF were included in study outcome measures. The study continues to benefit from never losing the perspective of the comprehensive ICF framework. Second, with regard to study implementation, OTs are familiar with standardized upper extremity motor assessments, as well as cognitive assessments from their clinical work. Having these perspectives are invaluable to inform the feasibility of research outcomes. The OTs specifically contributed to identifying a combination of outcome measures for UE motor function that are both meaningful and practical. This practical application of clinical practice and knowledge added to the efficiency of research testing which is critical for feasibility in the acute care setting. Third, performing clinical research on patients during the acute phase of stroke recovery poses challenges to a patient's limited physical, emotional, and mental endurance for tolerating activity. To this end, OTs' expertise at providing clinical care to patients with impaired arousal and attention allowed for discussion amongst research staff on how to best optimize participation in outcome assessments. Recommendations for environmental set-up and strategies to facilitate arousal and attention were also important contributions to efficiently and safely enable patient testing in the dynamic acute hospital setting.

## Successes, Challenges, and Strategies

There are many factors that contributed to the ongoing success of inpatient OTs participating in clinical research and data collection during acute hospitalization for stroke. These include the infrastructure of the inpatient occupational therapy department and hospital culture regarding OT's contribution to the multidisciplinary team, the value placed on research, interdisciplinary approach to stroke care and a strong relationship with academic rehabilitation science researchers. These factors are discussed below.

### Infrastructure

The size and structure of the MGH Inpatient Occupational Therapy Department contributes to the ability to have 1-year rotations on specific service areas, such as neurology, where OTs have exposure to sub-groups of patient populations over time. This model allowed for clinicians involved in the study to use their advanced clinical skills and knowledge to assist in developing guidelines for the research assessment battery. The advanced knowledge of stroke recovery and stroke unit systems allowed clinicians to problem solve research participation and coordination of care issues. There was a large enough pool of clinicians both interested in contributing to research and qualified to serve as study staff who joined the study to prevent burnout among study staff. The OT Clinical Specialist in neurology at MGH has a unique role which includes overseeing clinical practice, supporting professional development of staff and leading quality process improvement initiatives on the neurology service. The OT Clinical Specialist's clinical care expectations is only 70%, allowing for the flexibility in the remaining 30% time to participate in weekly research meetings and to develop roles and responsibilities for other OTs within the study. This role was imperative to maintaining successful operations both within the interdisciplinary research team as well as the research OT group.

### Shared Value of Research

There is a strong value placed on research at MGH as an institution as well as within the occupational therapy department. Research OTs volunteered their time to complete the assessments, often needing to extend their work day to meet their clinical productivity expectations. Although this highlights one of the significant challenges, it also demonstrates the powerful commitment of these OTs to research and the motivation to contribute to the evidence that promotes stroke recovery for patients. The MGH Inpatient Occupational Therapy Department has a strong relationship with the OT faculty at the MGH Institute of Health Professions. This academic-practice partnership blends clinical and research expertise to maximize OT involvement and contribution to this study. The research expert for stroke UE motor recovery on the occupational therapy faculty at the MGH Institute of Health Professions was critical to the development of training protocols. These training protocols ensured occupational therapy research staff demonstrated continued proficiency in standard administration of tests. The physician-scientists who had the vision to create the interdisciplinary research team are to be commended for their commitment to an approach that recognizes and values the contribution of each discipline in caring for patients with stroke. This emphasis on interdisciplinary research is critical for moving the rehabilitation field forward to optimize outcomes for individuals who experience stroke.

### Training

Before initiating data collection, there was dedicated time for training and educating clinicians on the overview of the research study and the various assessments being administered. The demands of training six clinicians to ensure proficiency in administering tests was spread across two four-hour sessions. These sessions primarily addressed the Upper Extremity Fugl-Meyer to ensure standardization of the assessment across clinicians (5–7). OT and non-OT members of the research team participated together in these sessions. Approximately 1 year after study launch, an inter-rater reliability session was held. A two-way mixed-effect model with single ratings and absolute agreement was used to assess the intra-class coefficient (ICC) on proximal/distal/and speed coordination aspects of the Upper Extremity Fugl-Meyer among study staff (both OT and non-OT members). The ICC was calculated to be 0.80 [95% CI from 0.43 to 0.99, *F*_(2, 13.5)_ = 49.5, *p* < 0.001], confirming prior reports of the high inter-rater reliability of the Fugl-Meyer (8, 9). A one-time hour-long training session covered testing for the Box and Blocks Test and Nine Hole Peg Test. Once clinicians began participating in data collection, it was important to establish a structure to ensure that the assessment burden was equally shared amongst clinicians as well as to ensure all participants would be systematically assigned. The OT team established a rotation system to minimize burnout and share the workload burden. This was helpful to provide structure; however, flexibility was key in the success of the system. If a clinician was not present or had other commitments limiting their ability to perform the assessments, we had a coverage system where another clinician would substitute.

### Communication

Critical to the sustainability of interdisciplinary research in an acute care setting is ongoing communication regarding issues impacting feasibility. Understanding the severity of participants' deficits as well as the trajectory of the acute hospital course are influential factors in determining discharge and timeline for test completion. One of the biggest challenges was coordinating testing time in the context of an acute hospitalization. In the acute care setting where the focus is on diagnostic work-up of the current stroke and future stroke prevention, research OTs were vying for valuable time along with the primary team, consultative services, testing, imaging and clinical therapy. Finding research time that did not compete with the clinical needs of the patient required deliberate communication to coordinate. Since this is also necessary in clinical care coordination, we were able to use systems already available for quick and efficient communication. For example, a unique feature that our clinicians have access to are hospital-based cell phones where they can individually call and text nurses and other therapists to discuss plans for the day regarding the participant. In the electronic medical record, the therapists can view the participant's current location and thus avoid traveling to perform testing when participants are off the floor at a test or procedure.

### Efficiency

Balancing time to perform the study tests with clinical efficiency was a substantial issue. The OTs involved in the study all had busy, full-time clinical commitments. Many of the clinicians were on different rotations (outside of stroke) during their time on the study, which brought up challenges for coordinating care as they were often treating patients on services located in different buildings throughout the hospital. Study staff found that with increased experience performing the assessment battery, their proficiency in administering the assessments improved which had a direct and positive impact on efficiency. Another challenge to efficiency is the preparation prior to and just after test administration, which includes gathering/putting away testing materials, scoring and inputting results, environmental set-up and positioning needed to maximize participation. To combat this challenge, we utilized exercise physiology students and our occupational therapy aide to assist with care coordination, environmental set-up/clean up, gathering/putting away testing materials and electronic entry of test scores.

### Client Factors

Given the often fluctuating medical status of acute stroke patients and variability of post-stroke deficits, OTs with previous experience working with this complicated and heterogeneous population of patients were critical to successful data collection. In the early stages of recovery, a participant's cognitive status can often fluctuate and may interfere with their ability to effectively engage in assessments. At times, some participants' deficits in arousal, attention, and short-term memory required graded cueing or arousal stimulation. In addition, visual attention and processing, spatial awareness, praxis, postural control, and motor deficits might require adaptations to the environment and/or positioning of the patient. Participant fatigue, especially after stroke, is also an important factor influencing research testing. Having worked specifically with the stroke population for at least a 1-year rotation prior to joining the study, our research OTs could efficiently and effectively provide these adaptations as well as verbal and visual cues to maximize the patient's ability to perform the desired movement patterns for research testing. It is important to understand stroke impairments' effects on test performance and scoring criteria to maintain fidelity to testing instructions, maintain inter-rater reliability and document any interventions that may have affected these. OT study staff also utilized their advanced understanding and knowledge to assess patient's appropriateness for engaging in the assessment battery and recognizing when timing may improve test performance. If participants reported fatigue, study staff would terminate research testing and return later to complete assessments. Flexibility here was again key. Appreciating and understanding the acuity and fragility of this population in the acute hospital setting impacts the many factors that make research in this setting feasible.

## Recommendations for Future Interdisciplinary Stroke Research

To advance stroke rehabilitation research and approaches to improve recovery after stroke, it is clear that an interdisciplinary approach that addresses all domains of the ICF and spans the post-acute care continuum must be used. Stroke recovery research, starting in the acute phase after stroke, is challenging due to the need for coordination between research staff with clinical teams as well as the complexity and medical needs of acute stroke patients. OTs have distinct expertise in ICF domains as well as in stroke client factors and can be invaluable assets to the research team. Due to the heterogeneity of stroke, large sample sizes are needed to address important research questions; therefore, challenges identified must be addressed in order to support ongoing, multi-site data collection. This perspective is a call to action to the field for stroke recovery researchers to integrate clinical OT perspectives into ongoing research and for OTs to participate and lend their perspectives to ongoing studies. A summary of the perspectives of MGH acute care OTs on stroke recovery research is outlined in Box 1. Future studies may seek to establish a standardized, stroke recovery assessment battery, deployed as the standard of care in occupational therapy across healthcare systems and further across the United States and internationally. Such a battery will overcome many of the current challenges that straddle the clinical-research divide and may advance some of our research insights into clinical evidence-based practice.

Box 1 **Summary of perspectives of acute care occupational therapists on stroke recovery research.**

- Stroke recovery research too often occurs in silos, focused on one domain of the ICF and one professional perspective.
- To advance stroke recovery research, all aspects of the ICF should be accounted for in research studies. Clinical occupational therapists, core members of the interdisciplinary stroke recovery team, have unique perspectives on stroke survivors' ability to engage in meaningful activities to promote functioning and quality of life.
- Occupational therapists with full-time clinical commitments participated in a physician-scientist led stroke recovery research study at Massachusetts General Hospital, becoming integral members of the multidisciplinary study team and shaping all aspects of the study from study design to data collection and analysis.
- Unique contributions of OTs to the stroke recovery research study include addition of outcome measures measuring different domains of the ICF beyond body-structure/function, valuable perspectives on the feasibility of outcome measures, and unique qualifications for day-day research data collection in a busy inpatient stroke unit.
- Factors contributing to the success of OTs becoming integral members of the stroke recovery research team include (1) the infrastructure of the OT department, (2) the value placed on research in both the department and the institution, (3) the interdisciplinary approach to clinical stroke care translated into research, and (4) a strong relationship with academic rehabilitation science researchers.
- Potential barriers to clinical OTs participating in research are (1) time required for research assessment training and maintaining proficiency in tests (inter-rater reliability) (2) coordinating time for research testing for patients in the context of a busy acute stroke hospitalization (3) study staff balancing time for research with a busy clinical schedule and (4) complex and rapidly changing medical status of stroke patients.
- Strategies for success to overcome these barriers include (1) designing flexibility into the research schedule (2) ensuring regular communication between research and clinical staff (3) utilizing students and aides to maximize research testing efficiency and (4) applying clinical knowledge of patient factors to maximize research assessments.

## Author Contributions

JR, JA, AC, KC, HT, NF, CS, DL, and AR contributed to initial concept and perspectives. JR, JA, KC, HT, NF, CS, and AR developed the initial version of the manuscript. KE, CS, JR, and DL critically revised the manuscript. AC and DL provided objective data. All authors read and approved the final manuscript.

### Conflict of Interest

The authors declare that the research was conducted in the absence of any commercial or financial relationships that could be construed as a potential conflict of interest.
